# Motivational strategies used by health care professionals in stroke survivors in rehabilitation: a scoping review of experimental studies

**DOI:** 10.3389/fmed.2024.1384414

**Published:** 2024-05-15

**Authors:** Júlio Belo Fernandes, Sónia Fernandes, Josefa Domingos, Cidália Castro, Ana Romão, Susana Graúdo, Gonçalo Rosa, Tânia Franco, Ana Patrícia Ferreira, Claudine Chambino, Bruno Ferreira, Susana Courela, Maria José Ferreira, Isabel Silva, Vera Tiago, Maria João Morais, Joana Casal, Silvia Pereira, Catarina Godinho

**Affiliations:** ^1^Egas Moniz Center for Interdisciplinary Research (CiiEM), Egas Moniz School of Health and Science, Almada, Portugal; ^2^Nurs* Lab, Almada, Portugal; ^3^Department of Nursing, Unidade Local de Saúde de Almada-Seixal, Hospital Garcia de Orta, Almada, Portugal; ^4^Department of Nursing, Unidade Local de Saúde de Almada-Seixal, ACeS Almada-Seixal, UCC Seixal, Seixal, Portugal

**Keywords:** stroke, motivational interviewing, patient compliance, rehabilitation, medicine

## Abstract

**Introduction:**

Cognitive and motor impairments are common among stroke survivors. Physical therapy is often used to improve the functional capacity of stroke survivors. However, limited adherence to rehabilitation programs is a challenge. Motivation plays a crucial role in the success of rehabilitation programs as it influences individual adherence to treatment and overall health outcomes. This review aims to identify current trends in motivational strategies used by healthcare professionals for stroke survivor rehabilitation.

**Methods:**

Following the framework developed by Arksey and O’Malley, a scoping review was conducted. We performed a literature search using MEDLINE, CINAHL, the Cochrane Central Register of Controlled Trials, Nursing & Allied Health, and MedicLatina databases.

**Results:**

A total of 906 papers were identified. After selecting and analyzing the articles, 17 papers were included in this review. Health professionals use various strategies to motivate stroke survivors. These approaches include establishing a therapeutic alliance, improving patients’ health literacy, defining realistic goals, fostering problem-solving skills, personalizing the rehabilitation program, showcasing success stories, utilizing persuasive techniques, offering encouragement and compliments, providing emotional support, and effectively managing symptoms.

**Conclusion:**

The knowledge gathered in this review can guide healthcare professionals in helping patients overcome barriers to rehabilitation, improve their motivation, and ultimately enhance their recovery outcomes.

## 1 Introduction

Stroke, a cerebrovascular event, poses a significant health concern characterized by its multifaceted risk factors, incidence rates, and adverse outcomes (1). Risk factors for stroke include a broad spectrum, including but not limited to hypertension, diabetes, obesity, smoking, excessive alcohol consumption, physical inactivity, unhealthy diet, and cardiovascular diseases such as atrial fibrillation and coronary artery disease (2). Stroke rates have been on the rise, primarily due to an aging population and improved survival rates following stroke events (3, 4), with the projected global age-standardized incidence rate of ischemic stroke for the year 2030 estimated to rise to 89.32 per 100,000 population (5). Despite medical advancements, stroke persists as the second leading cause of death and the third leading cause of disability worldwide, imposing substantial burdens on individuals, families, and healthcare systems (6).

Worldwide, 60% of stroke survivors experience permanent disabilities such as cognitive and motor disturbances, specifically affecting balance, coordination, proprioception, muscle tone, muscle strength, and gait (7, 8). The natural recovery occurs in 50% of patients, predominantly within the initial month, with limited progress observed beyond 6 months (9). Enhancing functional capacity ranks among the most prevalent aspirations for stroke survivors (10). Post-rehabilitation, many of these individuals can regain their functional abilities (11).

Physical therapy is a valuable therapeutic approach for stroke survivors. Healthcare professionals commonly recommend home exercise programs to help stroke survivors with clinical rehabilitation or self-management of long-term health conditions. Research strongly supports intensive, repetitive, task-focused practices (11). Adhering to the prescribed rehabilitation program and maintaining consistency can have long-lasting health benefits, such as improved physical function and a better quality of life (12). This adherence can significantly enhance stroke survivors’ well-being and reduce the healthcare system’s burden by fostering their independence (13). However, a well-documented challenge in rehabilitation is the limited adherence to exercise programs (14, 15).

The outcome of rehabilitation often varies based on individual motivation, despite similar medical conditions (16). Numerous studies have highlighted the crucial role of individual motivation in achieving positive health outcomes (16, 17). Adherence to a rehabilitation program is a tangible motivation indicator (18), while its absence hampers physical activity and post-stroke training (19). Integrating motivational strategies into the rehabilitation process can enhance individual adherence and improve their overall health outcomes (20, 21).

Previous studies show a positive link between exercise self-efficacy and initiating and maintaining physical activity, particularly in the early and middle stages of planned programs (22–24). Bandura et al. (20) define self-efficacy as an individual’s belief in their ability to accomplish specific goals or tasks, while motivation reflects the drive or desire to engage in such behavior. Self-efficacy is critical in shaping motivation, behavior, and performance.

Bandura’s social cognitive theory of self-efficacy (25) suggests that individuals’ beliefs about their capabilities to perform a specific task or achieve a particular goal can be influenced by four primary sources: (1) Mastery experiences involve personal experiences of mastering or completing tasks. When individuals succeed at something, their self-efficacy for similar tasks increases; conversely, failure can lower self-efficacy; (2) Vicarious experiences imply observing others, particularly those like oneself, successfully performing tasks. This boosts their self-efficacy because it suggests they can attain similar results through effort and learning; (3) Social persuasion involves receiving feedback, encouragement, or support from others. Positive feedback and persuasive communication can help individuals believe in their abilities to overcome challenges; and (4) Physiological and affective states refer to the emotional and physiological reactions experienced during task performance if an individual associates a particular task with high anxiety or stress levels, their self-efficacy may be lower.

Motivation is crucial in driving behavior change and adherence to rehabilitation programs. Motivational theories can provide healthcare professionals with valuable insights into designing and implementing effective interventions tailored to individual needs (26). Self-determination theory sheds light on the relationship between motivation and behavior. It posits that individuals are inherently motivated to pursue activities that fulfill three basic psychological needs: autonomy, competence, and relatedness (27). Empowering patients by involving them in decision-making regarding their treatment plans and goals in stroke rehabilitation can enhance their intrinsic motivation to engage in rehabilitation activities. Likewise, fostering a sense of competence by providing appropriate feedback and setting achievable goals can boost patients’ confidence and motivation.

Another relevant theory is the transtheoretical model of health behavior change, which suggests that behavior change occurs in stages and involves a series of cognitive and behavioral processes (28, 29). In stroke rehabilitation, understanding where patients are in their readiness to change can inform the selection of appropriate motivational strategies. For instance, patients in the pre-contemplation stage may benefit from raising awareness about the importance of rehabilitation. In contrast, those in the action stage may require support in overcoming barriers to adherence.

Challenges to adhering to rehabilitation programs exist, and prior studies show that interventions enhancing motivation can be effective (21, 30). By integrating insights from motivational theories into clinical practice, healthcare professionals can tailor their interventions to address the diverse needs of stroke survivors. Ultimately this will enhance adherence to rehabilitation programs and improve health outcomes. This comprehensive understanding of motivational strategies can inform the development of more effective and sustainable approaches to stroke rehabilitation (20). Therefore, this review aims to identify motivational strategies used by healthcare professionals during stroke survivor rehabilitation.

## 2 Methods

The researchers conducted a scoping review following the five-step framework initially developed by Arksey and O’Malley (31) and the recommendations of Levac et al. (32). These steps included:

1. Defining the research question(s)
2. Identifying relevant studies
3. Selecting the studies
4. Charting the data
5. Collating, summarizing, and reporting the results

The review involved an extensive literature search on the motivational strategies used by healthcare professionals in stroke survivors’ rehabilitation. The reporting adhered to the Preferred Reporting Items for Systematic Reviews and Meta-Analyses extension for Scoping Reviews (PRISMA-ScR) (33).

### 2.1 Stage 1: identifying research questions

The following research question was formulated employing the PCC framework (Population, Concept, and Context) to ensure the inclusion of all pertinent literature in this review: What motivational strategies (C) do healthcare professionals employ in the rehabilitation (C) of stroke survivors (P)?

### 2.2 Stage 2: identifying relevant studies

The researchers searched five databases: MEDLINE, CINAHL, the Cochrane Central Register of Controlled Trials, Nursing & Allied Health, and MedicLatina, covering 2003–2023. The search included publications in English, Portuguese, and Spanish. The final search was performed on June 28, 2023.

The PCC framework served as the foundation for developing the search strategy and establishing criteria for inclusion and exclusion (Table 1).

**Table 1:** Eligibility criteria.

Parameter	Inclusion criteria	Exclusion criteria
Population	Stroke survivors; Adults ≥18 years old.	Other health conditions besides stroke; Participants <18 years old.
Concept	Studies that explore motivational strategies developed by healthcare professionals.	Studies that do not address motivational strategies.
Context	Studies conducted in rehabilitation settings (e.g., acute, post-acute, long-term care, and home care).	Studies conducted in non-healthcare or non-rehabilitation settings.
Study design	Experimental and quasi-experimental trials and trial protocols.	Other type of studies.

Medical Subject Headings (MeSH) terms were employed in conjunction with Boolean operators to create the following search string: (Stroke) AND (Motivation OR Adherence OR Self Efficacy OR Patient Compliance) AND (Rehabilitation OR Exercise OR Physical Therapy Modalities OR Physiotherapy OR Physical therapy).

### 2.3 Stage 3: study selection

After removing duplicates, the titles and abstracts of each citation were independently screened by two researchers utilizing Rayyan, an AI-powered tool for systematic literature reviews. After obtaining relevant studies in full text, each researcher individually assessed each study to ensure agreement on whether it satisfied the inclusion/exclusion criteria. Any disparities in eligibility were resolved either through consensus or with the assistance of an additional researcher.

### 2.4 Stage 4: charting the data

Two reviewers conducted data extraction employing a standardized electronic data extraction form. The following data were extracted from each study:

- General data (author’s name, publication year, title, and country).
- Methodological data (study design and objectives).
- Results (strategies employed to motivate participants).

All authors underwent a thorough review and discussion of the final extraction chart.

### 2.5 Stage 5: collating, summarizing, and reporting the results

A data-driven thematic analysis, guided by Braun and Clarke’s framework (34), was employed to structure and synthesize the data. Two researchers individually examined the data, manually applying codes during the analysis process. Patterns and consistencies were systematically explored to determine prevalent themes from data based on Bandura’s framework (25).

A quality assessment or critical appraisal was not performed due to the objective of this review.

## 3 Results

**Figure 1:**
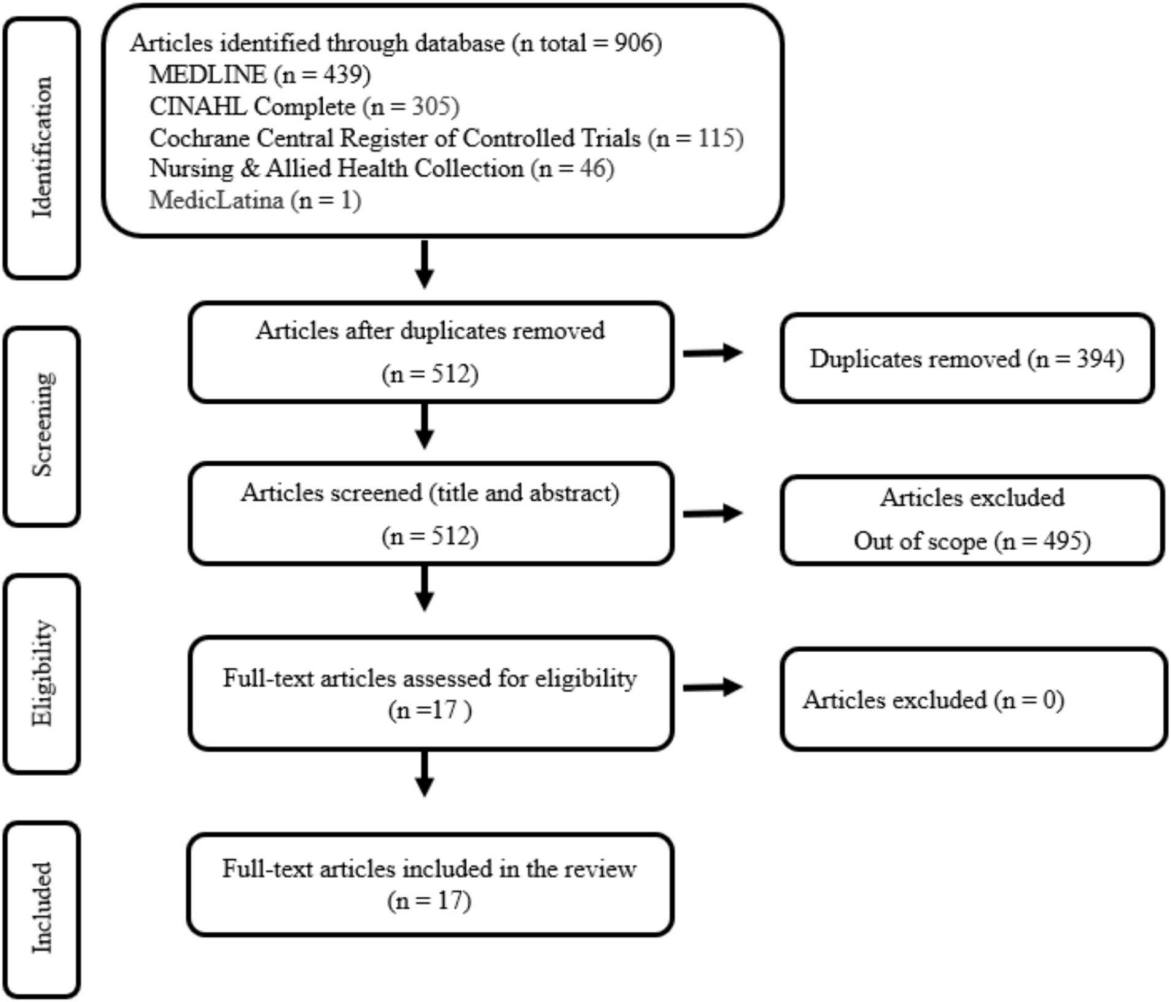
PRISMA flow diagram for study selection.

The initial search retrieved 906 studies (Figure 1), which were exported to Rayyan AI-powered tool for systematic literature reviews. After the duplicate removal, 512 titles and abstracts were screened for eligibility, of which 495 were excluded. The remaining 17 were read in full, meeting the inclusion criteria and being incorporated into this study.

This scoping review has enabled the identification of various strategies to motivate stroke survivors to adhere to the rehabilitation program.

Among the included studies (Table 2), five were conducted in Australia (35, 36, 42, 44, 45) two in China (47, 48), two in Israel (43, 50), one in the Netherlands (37) one in Norway (38), one in France (40) one in Denmark (39), one in Spain (41) one in Thailand (46), one in Ghana (49), and one in Sweden (51).

**Table 2:** Data extraction and synthesis.

Author/Year/Country	Study design	Aim	Interventions
Hoffmann et al. (35), Australia	Randomized controlled trial	To evaluate the effectiveness of providing stroke patients with computer-generated tailored written information.	Education practice (verbal discussions with health professionals)
			Establish participant’s informational needs
			Computer-generated, tailored written information
Graven et al. (36), Australia	Study protocol for a randomized controlled trial	To investigate the effectiveness of a client-centered, integrated approach to facilitating goal achievement and recovery in the first year post-stroke.	Written material (booklet and factsheets)
			Home visit
			Emphasize the “best effort”
			Verbal encouragement
			Telephone contact
			Verbal support
Tielemans et al. (37), The Netherlands	Randomized controlled trial	To investigate the effectiveness of a self-management intervention aimed at proactive coping for stroke patients and partners, compared with an education intervention.	Proactive action planning
			Peer support
			Stroke education
			Handle negative emotions
Stock et al. (38), Norway	Randomized controlled trial	To investigate to what degree patients adhered to a modified constraint-induced movement therapy protocol and to explore factors associated with the results.	Goal setting
			Daily Schedule
			Home diary
			Behavioral contract
Pallesen et al. (39), Denmark	Study protocol for a randomized controlled trial	To investigate the effect of a novel self-management intervention to support elderly people after stroke.	Introduction meeting before discharge
			Establish a good relation
			Map patients’ social network
			Supportive meetings/visits or telephone calls
			Mentors coach stroke survivors and their informal caregivers
			Encourage them to be active in decision-making
Chaparro et al. (40), France	Study protocol for a randomized controlled trial	To evaluate the effects of a home-based physical activity incentive and education program (Ticaa’dom) on functional capacity in subacute stroke patients.	Educational diagnosis
			Selection of physical activities
			Goal setting
			Telephone calls
			Encourage regular physical activity
			Inquire about physical activity
			Home visit
Gual et al. (41), Spain	Study protocol for a randomized controlled trial	To assess the impact of MI, as a complement to standard geriatric rehabilitation, on functional improvement at 30 days after admission, compared to standard geriatric rehabilitation alone, in persons admitted to geriatric rehabilitation after a stroke.	Motivational Interviewing
			Identify the person’s particular strengths, abilities, and efforts
			Goal setting
			Adapt the plan to the improved abilities
			Reflective listening
			Inform and advise
			Create engagement
			Explore preferences, values, goals, and knowledge and expectations about stroke rehabilitation and recovery
			Evoke the person’s strengths and abilities
			Follow-up and reinforcement
			Debrief with other professionals
Minshall et al. (42), Australia	Randomized controlled trial	To evaluate the effectiveness of a novel psychosocial intervention designed to improve health outcomes in both groups.	Structured workbook
			Professional facilitators worked with patients on an individualized basis
			Problem-solving
			Stress management
			Goal setting
Harel-Katz et al. (43), Israel	Randomized controlled trial	To evaluate the feasibility and effectiveness of the IPASS adapted for an Israeli population of individuals admitted to a day-rehabilitation center after stroke.	Learn and practice self-management skills
			Problem-solving and decision-making
Lin et al. (44), Australia	Study protocol for a randomized controlled trial	To determine the effectiveness of nurse-led health coaching for stroke survivors and primary caregivers in hospital-to-home transition care in Chongqing, China.	Goal setting
			Personal action plans
			Therapist demonstrates self-care and other skills required
			Individualized physical exercise plan
			Coaching Diary
			Stroke education
			Phone support and face-to-face meetings
			Encourage participants to discuss concerns and problems arising
			Develop problem-solving skills
			Introduce resources
			Reinforce self-care knowledge and skills
			Motivate to self-monitor progress and outcomes toward care goals
Brauer et al. (45), Australia	Randomized controlled trial	To determine, in individuals undergoing rehabilitation after stroke, if 8 weeks of high-intensity treadmill training embedded in self-management education (i) results in more physical activity than usual physiotherapy gait training and (ii) is more effective at increasing walking ability, cardiorespiratory fitness, self-efficacy, perception of physical activity, participation, and health-related quality of life as well as decreasing cardiovascular risk, and depression, at 8 and 26 weeks.	Detailed workbook
			Encourage self-monitoring of physical activity
			Goal setting and goal review
			Formulate action plans
			Stroke education
			Behavioral instruction
			Self-monitoring
			Feedback
			Problem-solving
			Inform participants about the consequences of their action
Tangnitipong et al. (46), Thailand	Randomized controlled trial	To assess the effectiveness of a home-based coping enhancement program on psychological well-being and adherence to rehabilitation in stroke survivors in northern Thailand	Goal setting
			Action plan
			Stroke education
			Booklet and video
			Stress management
			Problem-solving
			Emotional support
			Social support
			Skill building
			Verbal reinforcement and advice
			Telephone calls
Zhang et al. (47), China	Study protocol for a randomized controlled trial	To investigate the effectiveness of coaching-based teleoccupational guidance (CTG) for homebased stroke survivors and their caregivers.	Establish equal, friendly, and mutual trust relationships
			WeChat group
			Goal setting
			Individualized problem-solving plan
			Motivational interview
			Emotional support
			Express empathy
			Assess participants needs
			Individualized content education
			Coaching video session
Zhang et al. (48), China	Randomized controlled trial	To investigate the feasibility and effectiveness of a 3-month coaching-based eleoccupational guidance (CTG) program for home-based stroke survivors and their family caregivers.	Goal setting
			Coaching relationship
			Care plan
			WeChat coaching video sessions
Osei et al. (49), Ghana	Study protocol for a randomized controlled trial	To test the feasibility of a nurse-led telerehabilitation intervention in improving self-efficacy among stroke survivors.	Video calls on WhatsApp or phone calls
			Guide patients to establish a structured routine
			Stroke education: based on participant’s and caregiver’s learning needs
			Assess and support the emotional status
Adamit et al. (50), Israel	Randomized controlled trial	To examine the effectiveness of FaCoT compared to a control group to improve self-efficacy, behavior, and emotional status (secondary outcome measures).	Goal setting
			Planning and decision-making were taught and practiced
			Success log
			Providing the participants with a sense of success
			Highlight the experiences of success
			Using case studies
			Positive therapeutic language and positive feedback
			Emphasize participants’ personal abilities, efforts, and progress
			Uncover hidden symptoms and linking them to their function post-stroke
			Raise awareness regarding physiological and emotional symptoms
Thurston et al. (51), Sweden	Study protocol for a randomized controlled trial	To test the feasibility, acceptability, and preliminary effects of a mobile Health (mHealth) version of the i-REBOUND program for promoting physical activity in people post-stroke or TIA living in Sweden.	Prescription of exercise through mobile app videos
			Individual counseling
			Assess exercise preferences and barriers to physical activity
			Goal setting
			Educational videos
			Self-monitoring (STAAR app using an activity diary)
			Video meetings

Health professionals use several interventions to motivate stroke survivors to adhere to the rehabilitation program. These interventions were integrated into the four sources of influence on self-efficacy defined by Bandura et al. (25), as presented in Table 2 and detailed below.

### 3.1 Mastery experiences

Bandura’s theory (25) suggests that mastery experiences stem from an individual’s direct encounters with success or failure within a specific task or situation. These past performances can serve as potent influences in cultivating self-efficacy. In this context, several studies have been identified that applied strategies such as the establishment of a therapeutic alliance (39, 41, 44, 47, 48, 50), enhancing health literacy (35–37, 40–42, 44–49, 51), setting achievable goals (38, 40–42, 44–48, 50, 51), personalizing rehabilitation programs (37–42, 44, 46–49, 51), and honing problem-solving skills (39, 42–47, 50, 51).

In implementing these strategies, healthcare professionals employ specific interventions to establish a therapeutic alliance founded on equal, friendly, and mutual trust. This approach seeks insight into individuals’ unique qualities and discerns their needs. Healthcare professionals can use this knowledge to help patients set specific and relevant short-, medium-, and long-term goals, develop problem-solving skills, and customize exercise programs to fit their needs and abilities. This approach promotes consistent engagement in physical activities and encourages patients to make exercise a regular part of their lifestyle.

Researchers employed educational methods tailored to their learning needs to improve the comprehension of stroke survivors and caregivers regarding stroke and recovery. Several studies have supplemented verbal information with leaflets, booklets, and factsheets (35, 36, 46) to facilitate this process. One study used computer-generated personalized written information to create a tailored booklet based on the patient’s informational needs (35). Furthermore, Tangnitipong et al. (46) and Thurston et al. (51) complemented stroke education with educational videos (Table 3).

**Table 3:** Strategies and interventions.

Domain	Strategies	Intervention
Mastery experiences	Therapeutic alliance	Introduction meeting
		Create engagement with the stroke survivor and their caregiver
		Gain insight into their lives before the stroke
		Express empathy
		Establish an equal, friendly, and mutual trust relationship
		Build coaching environment
	Health literacy	Identify patient’s and caregiver’s learning needs
		Individualize content education
		Help patients reach their conclusions about the information provided
		Provide alternative options to incorporate physical activity in their life
		Provide written material and educational videos
	Set achievable goals	Explore patients’ preferences, values, and expectations
		Assess participants needs
		Set specific and patient-centered sort-medium and long-term goals
		Conferrer with patients to adjust goals at different stages of rehabilitation
	Problem-solving method	Develop problem-solving skills based on patients’ needs
		Develop a structured and individualized problem-solving plan
		Structured follow-ups
	Personalize the rehabilitation program	Assess the patient’s abilities, preferences, and needs
		Develop an individually tailored exercise program
		Debrief with other professionals to tailor and adjust the program
		Discuss the program and demonstrate skills
		Guide patients to establish a structured routine
		Prescribe individual exercise programs through mobile app videos
		Adapt the plan to the improved abilities
		Tailor the instruction and program to make the task understandable
		Calendar of daily exercise activities
		Physical activity diary
Vicarious experience	Sharing cases	Use everyday scenarios of case studies
		Provide instructions on how to perform the behavior
		Demonstrate self-care and other skills required by patients
		Educational videos
Verbal persuasion	Persuasion	Describe the benefits of physical activities
		Inform them about the consequences of their actions/inaction
		Educational videos
		Behavioral contract
		Home visit
		Telephone calls
		Apps to push notifications as reminders
	Encouragement and compliment	Positive therapeutic language
		Give verbal encouragement and compliment
		Emphasize participants’ personal abilities, efforts, and progress
		Assert that participants can self-manage
		Motivation interviewing
		Family involvement
		Success log
		Digital activity coaching system
Physiological and affective states	Emotional support	Encourage participants to discuss concerns and problems arising
		Identify barriers to participating in the rehabilitation program
		Identify strategies to overcome potential barriers
		Work with patients on an individual basis and offer flexible delivery times and modes
		Use the problem-solving method to address perceived barriers to participation in rehabilitation programs
		Assess and support the emotional status of patients and their caregivers
		Understand the extent of loss, recognize emotions and mood changes
		Improve patients’ self-efficacy to manage their emotional condition
		Handle negative emotions
		Stress management
		Family support
	Symptom management	Uncover hidden symptoms and link them to their function post-stroke
		Raise awareness about symptoms and the impact of these on their daily living
		Identify and manage stroke-related complications
		Improve participants’ self-efficacy to manage their medical condition

### 3.2 Vicarious experience

In various studies, vicarious experiences have played a crucial role (44–51). In this context, researchers have shared the success stories of other patients, thereby fostering confidence and motivation among individuals encountering similar challenges. By observing the achievements of others in similar situations, participants were expected to develop the determination needed to reach their own goals. Additionally, researchers provide clear instructions for desired behaviors, demonstrate essential self-care skills for patients, and use educational videos as informative resources.

### 3.3 Verbal persuasion

Various strategies directed toward persuasion and encouragement have been identified. Studies described the benefits of engaging in physical activities, informing individuals about the consequences of their actions (or inaction), and encouraging them to consider the costs and benefits of their behavior carefully (36, 38, 39, 41, 44–49, 51).

Moreover, educational videos have been used in this context, highlighting the health advantages of physical activity, particularly for secondary stroke prevention and overall well-being. Additionally, researchers have employed behavioral contracts, conducted home visits, made telephone calls, and utilized mobile apps equipped with push notifications to act as reminders for performing activities. These apps also provide feedback on the percentage of goal fulfillment over time.

In addition to these strategies, Zhang et al. (47, 48) introduced a unique approach by asking stroke survivors to create and share videos. These videos featured the practice of occupational exercises and participation in activities of daily living, among other relevant content.

Researchers implemented a comprehensive set of interventions to provide encouragement and compliments (36, 38–41, 46–48, 50, 51). These interventions encompassed positive therapeutic language and acknowledged and praised participants for their efforts. Furthermore, they provided verbal encouragement and compliments to boost participants’ confidence and motivation. Another aspect of this strategy involved emphasizing each participant’s unique personal abilities, efforts, and progress, instilling a sense of individual achievement and growth. Researchers encouraged participants to believe in their capacity to self-manage their rehabilitation process, promoting a sense of autonomy and self-efficacy.

Family involvement played a significant role, enlisting the support of the participant’s family to provide additional encouragement and a strong support network.

One study used a success log to record and celebrate participants’ successes and achievements throughout their rehabilitation journey to reinforce the sense of accomplishment. Zhang et al. (47, 48) also used digital activity coaching systems to offer ongoing guidance and support for physical activities and rehabilitation, leveraging technology to enhance motivation and progress.

### 3.4 Physiological and affective states

In physiological and affective states, participants were encouraged to discuss their concerns and problems, identify potential barriers to engagement, and create strategies to overcome these obstacles (37, 39, 42–49).

Moreover, problem-solving was employed to address perceived barriers to participation in rehabilitation programs (45, 51). Emotional assessment and support were integral, encompassing the evaluation and assistance of patients and their caregivers in managing their emotional well-being. Researchers sought to understand the extent of loss, recognize emotions and mood changes, and identify personalized coping strategies tailored to everyone’s needs.

The emotional support approach included strategies for managing negative emotions, dealing with stress, and getting family support. Under Symptom Management, researchers designed a set of interventions to address various aspects (39, 41–46, 49, 50). These interventions involved uncovering hidden symptoms and establishing connections with their post-stroke functionality. Participants were also aware of symptoms such as fatigue, cognitive impairments, low self-efficacy, and their impact on daily living. The strategy also included the identification and management of complications related to stroke. Moreover, participants were actively supported to enhance their self-efficacy in managing their medical condition and participating in rehabilitation programs.

## 4 Discussion

This review identified 72 interventions used by health professionals to motivate stroke survivors. Utilizing Bandura’s Theory (25) as the conceptual framework, the findings of this review were categorized and interpreted, grouping the interventions into 11 strategies.

The results of this review emphasize the significance of engaging the patient and their family right from the outset of stroke rehabilitation. Establishing a therapeutic alliance from the beginning is not merely a procedural formality but a foundational step with profound implications for rehabilitation. It acknowledges that the rehabilitation process is a collaborative journey, recognizing the patient as an active participant rather than a passive recipient of care, thereby enabling the early identification of the patient’s needs (52, 53). Early identification is instrumental in tailoring the rehabilitation process to each person.

Collaboratively setting achievable goals is another cornerstone of effective stroke rehabilitation. By involving stroke survivors and their families in this process, goals become more meaningful and aligned with the patient’s desires and capabilities. This enhances motivation and promotes a greater sense of ownership and commitment to the rehabilitation journey (31).

The development of personalized programs reflects the acknowledgment that each stroke survivor’s experience is unique, and their rehabilitation plan should be tailored accordingly (54, 55). These personalized plans address not only physical needs but also emotional and psychological requirements, promoting holistic recovery. In addition, personalizing rehabilitation programs ensures that the interventions are closely aligned with the patient’s needs, abilities, and aspirations (56). This tailored approach maximizes the chances of success and minimizes the risk of interventions that may be irrelevant or overwhelming (57). Stroke survivors often encounter a range of challenges during their recovery. By honing problem-solving skills, patients and their families can develop strategies to overcome obstacles as they arise (55). This proactive approach empowers patients to navigate the complexities of rehabilitation more effectively.

Sharing success stories of other patients who have faced similar challenges is a powerful motivational strategy. It allows stroke survivors to identify with the achievements of their peers, fostering a sense of confidence and hope. These inspiring cases demonstrate that recovery is possible, which is fundamental for patient motivation (58).

Persuasion and encouragement are pivotal in motivating stroke survivors to actively engage in their rehabilitation journey. In addition to face-to-face contact and phone or video calls, several studies use educational videos focusing on the health benefits of physical activity for secondary stroke prevention, and overall health stands out as a particularly influential strategy (46–48, 51). Previous studies also show that educational videos can be a powerful persuasion tool, providing concrete evidence of the positive outcomes they can achieve through their efforts (59).

Behavioral contracts can be another persuasive intervention that should be taken into consideration. These contracts formalize the patient’s commitment to their rehabilitation goals. Previous studies verified that by signing such a contract, patients commit to the prescribed activities and interventions. This formalized commitment can significantly boost motivation and accountability, as patients feel responsible for honoring the agreement (60).

The study by Thurston et al. (51) used a mobile application to push notifications. These applications serve as continuous reminders to patients to carry out their rehabilitation activities. Moreover, they can deliver a concrete sense of progress by providing feedback on the goal attainment percentage over time.

In addition to persuasion, several studies used encouragement and compliments to boost patients’ self-esteem and self-efficacy. When healthcare professionals acknowledge and praise patients’ efforts, it can enhance their confidence and motivation to continue striving. It reinforces progress and successes along the journey (17, 61).

Providing emotional support and effective management of post-stroke symptoms is critical, as survivors often face significant symptoms and emotional challenges (62, 63). Encouraging open discussion of concerns, identifying post-stroke symptoms and emotional barriers, and providing flexible support can help patients cope with their condition, enhancing their overall well-being.

The results of this review indicate a growing trend in incorporating new technologies to improve stroke survivors’ adherence to rehabilitation programs. Interestingly, several studies (64, 65) have already embraced mobile applications as an integral part of their interventions to educate, monitor, and counsel patients. This underscores the increasing awareness of the potential of digital technologies to enhance the effectiveness and accessibility of rehabilitation.

Mobile apps hold the potential to offer a range of benefits, including the delivery of personalized information, the ability to monitor patient progress, the provision of real-time reminders and feedback, and making rehabilitation more accessible, especially in situations where in-person therapy may be limited (66, 67).

As technology continues to evolve and become more accessible, it is reasonable to anticipate that more studies and rehabilitation programs will adopt and adapt these tools.

Overall, the review pioneers a motivational approach to stroke rehabilitation. Stroke rehabilitation is a complex journey that demands physical recovery and patient commitment. Motivation is pivotal in ensuring stroke survivors actively engage in and adhere to their rehabilitation programs, significantly influencing their recovery outcomes (68). This review fills a crucial gap by being the first to systematically identify and categorize motivational strategies healthcare professionals use to support stroke patients during their rehabilitation process. It provides an overview of the different techniques that can be used to increase motivation in this group.

From a practical perspective, the study’s findings offer actionable insights for healthcare professionals involved in stroke rehabilitation. The study equips health professionals with a toolkit to tailor interventions to individual patient needs by delineating various motivational strategies. For instance, the emphasis on establishing a therapeutic alliance underscores the importance of building rapport and trust with patients from the outset, laying a solid foundation for collaborative goal-setting and personalized care plans. Similarly, incorporating technology, such as mobile applications, presents tangible opportunities to enhance stroke survivors’ engagement and adherence through real-time feedback, reminders, and educational resources.

Furthermore, identifying specific interventions, such as sharing success stories and providing emotional support, offers practical guidance on fostering motivation within rehabilitation settings. By leveraging these strategies, healthcare professionals can create supportive environments that empower stroke survivors to participate actively in their recovery journey, ultimately improving rehabilitation outcomes.

Theoretically, the study contributes to the ongoing discourse on self-efficacy and motivation within the context of health behavior change. Bandura’s Theory of self-efficacy (25) serves as a theoretical framework, elucidating how mastery experiences, vicarious experiences, verbal persuasion, and physiological/affective states influence individuals’ beliefs in their ability to achieve desired outcomes. By mapping the identified interventions onto these sources of influence, the study provides theoretical validation for their effectiveness in enhancing self-efficacy and motivating behavior change among stroke survivors.

Moreover, the study underscores the dynamic interplay between individual, interpersonal, and environmental factors in shaping motivational processes. The emphasis on collaborative goal-setting, family involvement, and personalized care highlights the socioecological nature of motivation, wherein interactions between individuals and their social contexts influence motivational outcomes. This aligns with contemporary behavior change theories, emphasizing the reciprocal determinism between personal factors, behavior, and environmental influences (27–29).

The review fosters a more nuanced understanding of motivational processes in stroke rehabilitation by bridging practical insights with theoretical frameworks. It underscores the importance of adopting holistic approaches that consider individual needs and broader socioenvironmental factors in promoting sustained behavior change and optimal recovery outcomes. Furthermore, this review provides solid evidence to inform the development of a specific care pathway for motivating stroke survivors. By integrating the identified strategies, best practices, and clinical recommendations, a care pathway can guide healthcare professionals in addressing patient motivation throughout the post-stroke rehabilitation process.

A well-designed care pathway can offer a systematic framework to ensure that motivational interventions are consistently personalized and applied to each patient’s needs and preferences (69). Additionally, by integrating appropriate outcome assessment measures, a care pathway can enable continuous monitoring of patient progress and adaptation of interventions as needed over time (70, 71).

By developing a patient-centered care pathway focused on motivation, healthcare providers can maximize the potential for stroke survivors’ recovery, promoting more consistent and enduring adherence to the rehabilitation process. This can significantly improve patients’ physical, cognitive, and emotional functionality and overall quality of life.

Furthermore, a motivation-focused care pathway can help optimize healthcare resources, minimizing the time and costs associated with treating complications resulting from a lack of adherence to rehabilitation. By promoting a proactive and preventive approach to post-stroke management, a care pathway can reduce hospital readmissions and improve long-term outcomes for stroke survivors.

### 4.1 Strengths and limitations

The findings from this review can have significant implications for future research, potentially serving as the groundwork for developing tailored care pathways that motivate patients to adhere to rehabilitation programs. This approach could enable healthcare professionals to integrate these interventions into daily practice effectively. However, it is important to acknowledge several limitations in this research. Firstly, the exclusive focus on experimental studies may introduce a bias toward strategies primarily employed by researchers rather than those commonly used by healthcare professionals in their routine care. Secondly, the restriction of databases might have excluded relevant studies. Lastly, including only English, Portuguese, and Spanish papers opens the possibility that studies in other languages could have influenced the review’s outcomes differently.

## 5 Conclusion

This review identified several strategies used by healthcare professionals to motivate stroke survivors to engage in rehabilitation programs. These strategies involve establishing a therapeutic alliance, enhancing patients’ health literacy, setting attainable goals, tailoring rehabilitation programs, employing problem-solving techniques, sharing case studies, utilizing persuasion, offering encouragement and compliments, and providing emotional support and symptom management. This research has a unique focus on motivation in stroke rehabilitation and highlights its importance in enhancing the quality of care and outcomes for stroke survivors. It contributes valuable insights that can positively impact the lives of those recovering from stroke and assist healthcare professionals in delivering more effective rehabilitation services.

## Data availability statement

The raw data supporting the conclusions of this article will be made available by the authors, without undue reservation.

## Author contributions

JF: Conceptualization, Data curation, Formal analysis, Investigation, Methodology, Project administration, Supervision, Visualization, Writing – original draft, Writing – review & editing. SF: Data curation, Formal analysis, Methodology, Writing – original draft, Writing – review & editing. JD: Writing – original draft, Writing – review & editing. CCa: Writing – original draft, Writing – review & editing. AR: Writing – original draft, Writing – review & editing. SG: Writing – original draft, Writing – review & editing. GR: Writing – original draft, Writing – review & editing. TF: Writing – original draft, Writing – review & editing. AF: Writing – original draft, Writing – review & editing. CCh: Writing – original draft, Writing – review & editing. BF: Writing – original draft, Writing – review & editing. SC: Writing – original draft, Writing – review & editing. MF: Writing – original draft, Writing – review & editing. IS: Writing – original draft, Writing – review & editing. VT: Writing – original draft, Writing – review & editing. MM: Writing – original draft, Writing – review & editing. JC: Writing – original draft, Writing – review & editing. SP: Writing – original draft, Writing – review & editing. CG: Writing – original draft, Writing – review & editing.
